# The transcript catalogue of the short-lived fish *Nothobranchius furzeri* provides insights into age-dependent changes of mRNA levels

**DOI:** 10.1186/1471-2164-14-185

**Published:** 2013-03-16

**Authors:** Andreas Petzold, Kathrin Reichwald, Marco Groth, Stefan Taudien, Nils Hartmann, Steffen Priebe, Dmitry Shagin, Christoph Englert, Matthias Platzer

**Affiliations:** 1 Genome Analysis, Leibniz Institute for Age Research – Fritz Lipmann Institute, Beutenbergstr. 11, Jena 07745, Germany; 2 Molecular Genetics, Leibniz Institute for Age Research – Fritz Lipmann Institute, Beutenbergstr. 11, Jena 07745, Germany; 3 Systems Biology, Leibniz Institute for Natural Product Research and Infection Biology - Hans-Knöll-Institute, Beutenbergstr. 11a, Jena 07745, Germany; 4 Evrogen Ru SJC, Milukho-Maklaya 16/10, Moscow 117997, Russia; 5 Shemyakin and Ovchinnikov Institute of Bioorganic Chemistry, Milukho-Maklaya 16/10, Moscow 117997, Russia

**Keywords:** *Nothobranchius furzeri*, Model fish species, Ageing, Transcriptome assembly, Transcript catalogue, Gene expression, RNA-seq

## Abstract

**Background:**

The African annual fish *Nothobranchius furzeri* has over recent years been established as a model species for ageing-related studies. This is mainly based on its exceptionally short lifespan and the presence of typical characteristics of vertebrate ageing. To substantiate its role as an alternative vertebrate ageing model, a transcript catalogue is needed, which can serve e.g. as basis for identifying ageing-related genes.

**Results:**

To build the *N. furzeri* transcript catalogue, thirteen cDNA libraries were sequenced using Sanger, 454/Roche and Solexa/Illumina technologies yielding about 39 Gb. In total, 19,875 protein-coding genes were identified and annotated. Of these, 71% are represented by at least one transcript contig with a complete coding sequence. Further, transcript levels of young and old fish of the strains GRZ and MZM-0403, which differ in lifespan by twofold, were studied by RNA-seq. In skin and brain, 85 differentially expressed genes were detected; these have a role in cell cycle control and proliferation, inflammation and tissue maintenance. An RNA-seq experiment for zebrafish skin confirmed the ageing-related relevance of the findings in *N. furzeri*. Notably, analyses of transcript levels between zebrafish and *N. furzeri* but also between *N. furzeri* strains differed largely, suggesting that ageing is accelerated in the short-lived *N. furzeri* strain GRZ compared to the longer-lived strain MZM-0403.

**Conclusions:**

We provide a comprehensive, annotated *N. furzeri* transcript catalogue and a first transcriptome-wide insight into *N. furzeri* ageing. This data will serve as a basis for future functional studies of ageing-related genes.

## Background

Invertebrate model species like yeast, worm and fly have been successfully used to study the genetic background of lifespan determination and ageing, leading to the identification of a number of relevant genes and pathways [1]. Although several of these pathways are conserved across taxa [2-4], reproducing the results in vertebrate species has not always been possible. A major drawback of vertebrate models like mouse or zebrafish is their relatively long lifespan of up to five years, which makes the validation of results obtained in invertebrates time-consuming and expensive. Alternative vertebrate model organisms are needed that allow validation of known and discovery of potentially new ageing-related pathways/genes within a reasonable time frame.

In 2005, the Turquoise killifish, *Nothobranchius furzeri*, was proposed as a new model for studies of ageing [5]. *N. furzeri* inhabit temporary ponds in South-East Africa and were first collected in 1968 in the Game Reserve Gona Re Zhou (GRZ) in Zimbabwe. Due to the seasonal nature of the habitat, this species shows an annual life cycle in which hatching, growth and reproduction happen during the wet season and fertilised eggs survive the dry season in the dry mud by entering into diapause [6]. Consequently, the lifespan of African *N. furzeri* populations is limited by the duration of the wet season, which lasts up to several months depending on location. Remarkably, the short-lived phenotype has been retained in captivity despite unlimited water and food supply, and *N. furzeri* laboratory strains originally derived from dry/humid habitats differ in lifespan [7]. For example, the GRZ strain, which stems from a dry habitat, has a maximum lifespan (10% survivorship) of 12-16 weeks [8,9]. The more recently isolated strain MZM-0403, originally collected in a humid habitat in Southern Mozambique (MZM), has a maximum lifespan of 25-32 weeks [7], which still is exceptionally short compared to other vertebrate species. Despite their short lifespan, the fish show typical ageing-related phenotypes such as physiological / cognitive decay and expression of ageing-related biomarkers [10].

Over past years, genetic and genomic resources for *N. furzeri* have been generated. A first insight into cytogenetic and genomic characteristics, including karyotype (19 chromosomes, 2n = 38), genome size (1.6–1.9 Gb) and composition (45% repeats), phylogeny (among fish with a sequenced genome available, medaka is the closest relative) were provided by our group [9]. Further, genetic variability in GRZ (highly inbred) and MZM-0403 (resembling a wild population) was assessed and a genetic linkage map, based on microsatellites and gene-associated single nucleotide variations, was created and used to identify several quantitative trait loci affecting lifespan [11]. On the other hand, sequence resources are still limited, i.e. a search for *Nothobranchius furzeri* in NCBI Entrez lists 13 genes, 279 nucleotide and 236 protein entries (NCBI Entrez, 18/02/13). Here, we set out to build a comprehensive, high-quality, annotated *N. furzeri* transcript catalogue. We used the catalogue in a pilot RNA-seq experiment aimed at identifying ageing-related genes, i.e. genes differentially expressed in the short- and longer-lived *N. furzeri* strains GRZ and MZM-0403 at young and old age.

## Results

### Assembly of the *N. furzeri* transcript catalogue

Transcriptome sequencing of GRZ and MZM-0403 using Sanger, 454/Roche and Solexa/Illumina technology yielded ~39 Gb in total (Table 1). To account for the different sets of raw sequence data (in the following referred to as reads), the transcriptome assembly was done in two steps: First, Sanger and 454/Roche reads were assembled with a classical overlap-layout-consensus strategy [12]. Three programs were tested: *The Gene Indices Clustering tools* (TGICL, [13]), *Newbler*[14] and *Program for Assembling and Viewing ESTs* (PAVE, [15]). PAVE produced the best assembly with respect to all assembly metrics (Table 2). Therefore, in the second step, the PAVE contigs were used as backbone for assembling Solexa/Illumina reads using an iterative procedure (see Methods). The final assembly resulted in 213,621 contigs with an average length of 1,183 bp (Table 2). Contigs ≥ 1 kb comprised 72% of the assembly. Of these, 230 contigs were larger than 10 kb.

**Table 1:** ***N. furzeri *transcriptome libraries and sequencing methods**

**Library**	**Specimens used for RNA-isolation**	**Sequencing technology**	**Number of reads**	**Average length (bp)**	**Total length (Mb)**
1	one male GRZ, 9 weeks, whole body; normalised^a^	Sanger	131,808	734	97
2	one male GRZ, 10 weeks, whole body; normalised^a^	454/Roche GS-FLX	751,885	223	168
3	one male GRZ, 10 weeks, whole body	454/Roche GS-FLX	415,426	225	94
4	six male and female GRZ, 8 weeks, whole body; normalised^b^	454/Roche GS-Titanium	2,351,236	369	869
5	three GRZ, 1 week, whole body	Solexa/Illumina, 2 x 150 bp	89,730,910	151	6,774
6	five male GRZ, 5 weeks, skin	Solexa/Illumina, 2 x 101 bp	55,410,042	101	5,596
		Solexa/Illumina, 76 bp	33,947,044	76	2,579
7	five male GRZ, 5 weeks, brain	Solexa/Illumina, 2 x 101 bp	60,997,820	101	6,161
		Solexa/Illumina, 76 bp	36,117,265	76	2,744
8	three male GRZ, 14 weeks, skin	Solexa/Illumina, 76 bp	35,963,921	76	2,733
9	three male GRZ, 14 weeks, brain	Solexa/Illumina, 76 bp	27,369,310	76	2,080
10	four male MZM-0403, 5 weeks, skin	Solexa/Illumina, 76 bp	31,727,935	76	2,411
11	five male MZM-0403, 5 weeks, brain	Solexa/Illumina, 76 bp	34,720,289	76	2,638
12	five male MZM-0403, 31 weeks, skin	Solexa/Illumina, 76 bp	30,268,649	76	2,300
13	three male MZM-0403, 31 weeks, brain	Solexa/Illumina, 76 bp	31,423,327	76	2,388
			467,676,512		39,632

^a^Normalisation was done by Evrogen.

^b^Normalisation was done by Vertis.

**Table 2:** **Assembly metrics**^**a**^

		**First assembly **^**a**^		**Second assembly **^**b**^
**Method**	**TGICL**	**Newbler**	**PAVE**	**Iterative assembly procedure**
**Input datasets**	Sanger, 454/Roche	Sanger, 454/Roche	Sanger, 454/Roche	PAVE contigs, Solexa/Illumina
**Number of contigs** (≥ 300 bp)	134,225	141,973	118,795	213,621
**Total length of contigs** (≥ 300 bp)	85.1 Mb	78.1 Mb	86.9 Mb	252.9 Mb
**Average length of contigs**	634 bp	549 bp	731 bp	1,183 bp
**Median length of contigs**	474 bp	434 bp	495 bp	676 bp
**Maximum length of contigs**	9,221 bp	7,841 bp	9,241 bp	64,116 bp
**Number of large contigs** (≥ 1 kb)	15,516	11,756	23,534	79,035
**Total length of large contigs**	24.9 Mb	19.1 Mb	38.3 Mb	183.0 Mb

^a^ ‘First assembly’ shows the results of three assembly programs that were tested using Sanger and 454/Roche data. PAVE produced the best assembly with respect to total length, number of contigs and average contig length. ^b^ ‘Second assembly’: Solexa/Illumina data were assembled onto a backbone of PAVE contigs using an iterative assembly procedure outlined in Methods.

**Figure 1:**
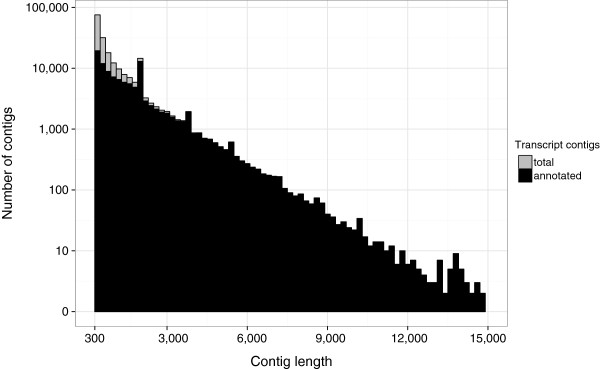
**Transcript contig length distribution. On the x-axis, contigs sorted according to their length in bins of 200 bp are shown.** Black and grey bars on the logarithmic y-axis indicate the fractions of annotated (black) and unannotated (grey) transcript contigs, respectively. The majority of all contigs larger than 1,000 bp is annotated.

Closer examination of the rather smooth contig length distribution revealed three prominent peaks at 2, 3.7 and 5.4 kb (Figure 1). Most likely, these peaks represent artefacts of the iterative assembly procedure in which contigs are fragmented into 2 kb-pieces with a 300 bp-overlap (see Methods). Respectively, the distance between the three peaks accounts for 1.7 kb, which equals to fragment length minus overlap. We hypothesised that a decent fraction of the artificial 2 kb input fragments could not be extended by the assembler due to repetitive motives at one (resulting in the 3.7 and 5.4 kb peaks) or both ends (2 kb peak). For confirmation, we searched for overlaps between the 2 kb contigs and the remaining transcript catalogue (BLASTn, e-value ≤ 10^-60^). Almost all contigs (9,026/9,033) showed significant overlaps (on average 379 bp, 96% identity), whereas 97% of the contigs with overlaps (8,793/9,026) showed multiple hits, with an average of 18 hits per contig. Similar results were obtained for contigs of the two other peaks (data not shown).

To quantify the impact of the three different sequencing technologies on our transcriptome assembly, all reads were mapped (Sanger and 454/Roche with Newbler; Solexa/Illumina with BWA [16]) onto the contigs of the final assembly. As a result, the vast majority of reads was mapped successfully (Sanger: 99%, 454/Roche: 92%, Solexa/Illumina: 97%). Sanger, 454/Roche and Solexa/Illumina reads covered about 20, 56 and 237 Mb, respectively, of the 253 Mb total contig length. Thereof, only 1, 7 and 178 Mb, respectively, were covered exclusively by either of the three technologies, showing the major impact of Solexa/Illumina on the final transcriptome assembly.

To assess the quality of the transcriptome assembly, transcript contigs were compared against a set of reference sequences obtained from the Ensembl genome annotation of medaka [17], the closest relative of *N. furzeri*. To avoid misinterpretation due to annotation errors of the medaka genome, we limited our analysis to the 1,750 medaka protein sequences with experimental support (Ensembl category “known”). All *N. furzeri* transcript contigs were compared with BLASTx (e-value ≤ 10^-20^) against the medaka protein sequences and evaluated for representation and coverage, assembly fragmentation and redundancy as well as putative chimerism. Altogether, 13,451 *N. furzeri* transcript contigs produced hits for 1,290 (74%) medaka proteins. Of these, 74% were covered by hits over ≥ 90% of their length. Moreover, for 67%, a hit by a single transcript contig was sufficient to achieve this coverage. To assess fragmentation, we determined the fractions of transcript contigs covering either the N-terminus (13%) or the C-terminus (16%) or neither one (64%) of the medaka protein. On the other hand, 7% were spanning the complete protein and included UTRs. In contrast to this relative low degree of fragmentation of the transcript catalogue, over 92% of the total identified protein sequence was hit by at least two transcript contigs with an average redundancy of nine contigs per aa. Finally, the BLASTx results facilitated identifying putative chimeric transcript contigs. To be marked as chimeric, a transcript contig was required to have two non-overlapping hits to different medaka proteins which meet the following criteria: e-value ≤ 10^-20^, coverage of both protein hits ≥ 75% and identity ≥ 50%. According to this definition, only 0.3% of the transcript contigs were identified as chimeric.

To identify transcript contigs derived from contaminating sources, tBLASTx searches (e-value ≤ 10^-20^) were performed against a database compiled from both fish-specific transcripts from Ensembl as well as non-fish transcripts from RefSeq [18]. For 1.9% of the contigs (4 Mb, median length: 531 bp), the best hit represented a non-fish species and the e-value of the next fish-specific hit was at least 10 orders of magnitude higher. Most hits were found for mammals (0.8%), followed by invertebrates (0.7%) and plants (0.3%). As the latter two may originate from tissue contamination by fish’s environment, those tagged as mammalian contamination represent rather laboratory artefacts acquired e.g. by the multistep cDNA normalization procedure prior Sanger sequencing (data not shown). The contigs were excluded from further analyses. Accordingly, 209,660 *N. furzeri* transcript contigs (249 Mb) were analysed further.

### Annotation of the *N. furzeri* transcript contigs

Annotation was based on BLAST searches against the following protein and nucleotide databases: (i) protein and transcript sequences of medaka, stickleback, tetraodon and zebrafish from Ensembl [17], (ii) human protein sequences from RefSeq [18], (iii) the UniProt database [19], (iv) the NCBI non-redundant protein database nr [20] and (v) fish-specific ESTs from NCBI UniGene [21]. BLAST parameters and e-value thresholds were defined for each database separately (Additional file 1: Table S1).

Of 209,660 *N. furzeri* transcript contigs, 122,177 (58%) had significant hits to at least one database (Table 3). The average length of these contigs was 1,604 bp, being considerably longer than for contigs with no hits (600 bp). A set of 75,091 (36%) transcript contigs had hits in all six databases.

**Table 3:** **BLAST based annotation of *N. furzeri *transcript contigs**

**Database**	**Contigs hit**	**Database entries hit**	**Avg. identity**	**Database entries used**	**Annotated contigs**
Ensembl fish proteins	98,941	38,355	73%	37,739 (81%)	92,857 (85%)
UniProt	98,053	42,361	71%	2,643 (6%)	3,425 (4%)
NCBI nr proteins	96,548	41,500	71%	956 (2%)	1,450 (1%)
Refseq human proteins	80,671	18,071	61%	305 (1%)	497 (1%)
NCBI UniGene transcripts	107,901	36,593	77%	3,206 (7%)	7,113 (6%)
Ensembl fish transcripts	98,099	39,301	77%	1,369 (3%)	3,690 (3%)
Total	122,177			46,218 (100%)	109,032 (100%)

For each transcript contig, BLAST hits were filtered and the best was selected for annotation. That way, 109,032 (52%) transcript contigs were annotated using 46,218 database entries. Of these, 21,728 (47%) were hit by more than one transcript contig, with an average of four per database entry. This reflects rather the redundancy than the fragmentation of the assembly (see above) and in respect to the protein hits provides an estimate of protein isoforms (see below). Subsequently, each transcript contig was assigned a gene symbol based on the information of the database entry used for its annotation. As a result, the *N. furzeri* transcript catalogue contains transcript contigs for 19,875 protein-coding genes. About half of all genes (10,392/52%) had multiple isoforms, with an average of three isoforms per gene.

**Figure 2:**
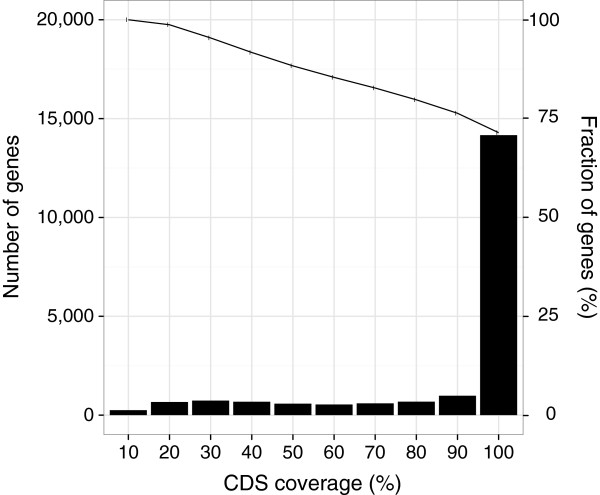
**Fractions of putative CDS represented in the longest transcript contig per *N. furzeri *gene.** Predicted CDS fractions were binned into deciles. The histogram bars show the number of respective transcript contigs (genes) per decile. The line shows the cumulative number. More than 70% of the protein-coding genes are represented by a transcript contig with a complete (> 90%) CDS.

The completeness of the *N. furzeri* proteins represented in the transcript catalogue was evaluated by analysing the proportion of coding sequence (CDS). Of 19,875 genes, 14,164 (71%) were represented by at least one transcript contig containing an almost complete CDS (> 90% in respect to that of the database entry used for annotation; Figure 2). Because CDS lengths generally differ largely and genes with smaller CDS are more likely to be complete, we performed a second analysis based on the total CDS length. To avoid counting redundant transcript contigs, only the longest transcript contig was selected for each gene. Altogether, the overall CDS of these contigs represented 29 Mb (83%) of 35 Mb obtained for the reference entries. In comparison to the four model fish species, the *N. furzeri* transcript catalogue represents 74% of zebrafish and 99% of medaka annotated CDS.

### Gene ontology of the *N. furzeri* transcript contigs

To strengthen the annotation of the *N. furzeri* transcript catalogue, contigs were assigned to Gene Ontology (GO, [22]) categories. The GO terms were obtained from the respective Ensembl and UniProt database entries used for the annotation of the transcript contigs. Of 109,032 annotated transcript contigs, 76,013 (72%) were linked to 340,452 GO counts. “Molecular Function” represented the largest class of assignments (70,305 contigs/ 166,476 GO counts), followed by “Biological Process” (49,972/108,336) and “Cellular Component” (40,551/65,640). After removing redundant counts, 6,796 unique GO terms were identified.

These GO terms were further mapped to GO-Slim terms, a set of 131 high-level functional annotations. In respect to the longest transcript contig per *N. furzeri* gene, 137,683 GO-Slim counts were assigned to 15,040 transcript contigs representing 124 unique GO-Slim terms (Additional file 2: Figure S1). Most frequent terms among the three GO domains were “metabolic process” (6,108 contigs, domain: biological process), “binding” (10,567 contigs, biological function) and “cell” (7,668 contigs, cellular component). All GO annotations are available through the “*Nothobranchius furzeri* Information Network transcriptome browser (see below).

### Comparison of *N. furzeri* transcriptome annotation to model fish genomes

BLASTx was used to compare the *N. furzeri* transcript catalogue to medaka, stickleback, tetraodon and zebrafish protein-coding genes currently available in Ensembl (Table 4). Among the *N. furzeri* transcript contigs, 46% had hits in at least one, 35% in all four and 4% in exclusively one species (Additional file 3: Figure S2). Protein conservation confirms that *N. furzeri* is more closely related to medaka, stickleback and tetraodon than to zebrafish [9].

**Table 4:** **BLASTx comparison of *N. furzeri *transcript contigs to the protein/gene annotation of four other fish genomes**

	**Medaka**	**Stickleback**	**Tetraodon**	**Zebrafish**
**Number of proteins/ genes annotated**	24,661 / 19,686	27,576 / 20,787	23,118 / 19,602	41,478 / 26,095
**Contigs with hits**	87,378	84,761	79,594	87,219
**Proteins/genes hit**	19,272 / 17,173	20,534 / 17,795	18,613 / 16,744	22,669 / 18,620
	78% / 87%	74% / 86%	81% / 85%	55% / 71%
**Average protein identity**	73.5%	74.6%	73.7%	67.5%
**Contigs with hits exclusively in this species**	2,791	1,256	531	2,791
**Proteins/genes hit**	914 / 914	690 / 690	356 / 356	942 / 942
**Average protein identity**	55.7%	57.0%	62.0%	49.5%

Since the comparison of gene numbers relies on the level of annotation and therefore can be biased, we evaluated the number of gene families, i.e. groups of genes with a common evolutionary ancestor. Gene family information for medaka, stickleback, tetraodon and zebrafish was obtained from the Ensembl Compara gene trees [23]. Zebrafish showed the highest number of gene families (8,241), followed by stickleback (7,663), medaka (7,501) and tetraodon (7,261). Based on the BLASTx results, we identified 7,489 gene families hit by at least one *N. furzeri* transcript contig. Moreover, for 85% (6,428 families), at least one of the four model fish species shared the same number of genes per family. These results indicate a completeness of the provided transcript catalogue comparable to that of model fish species for which an annotated genome is available.

### Duplicated *N. furzeri* genes

Teleost fish genomes underwent an additional round of genome duplication [24,25], compared to other vertebrates, and thus contain a high number of paralogs. Genes duplicated in *N. furzeri* were assessed in two steps. First, we identified those duplicated also in at least one of the model fish species. Towards this, for each *N. furzeri* gene the longest transcript contig was compared using BLASTx against Ensembl proteins of medaka, stickleback, tetraodon and zebrafish, and the Ensembl gene of the best hit was recorded. The corresponding Ensembl Compara gene tree was then used to find paralogs of this gene which are specific for teleost fish. If any of these paralogs were hit by another *N. furzeri* gene, then the two *N. furzeri* genes were considered as paralogous. By this, we identified among the 17,261 *N. furzeri* genes with BLASTx hits 4,498 paralogs grouped in 2,148 paralog families (2.1 paralogs per family, on average; Additional file 4). Second, among the remaining genes we searched redundant transcript contigs for indications of *N. furzeri*-specific paralogs using a clustering approach based on protein identities. For each gene, all transcript contigs were translated into proteins and aligned. Protein identities of these alignments were clustered based on a minimum distance of 10% between each cluster. For 1,249 genes more than one cluster was found, corresponding to a total of 2,619 (2.1 contig clusters per gene, on average; Additional file 4). Overall, this indicates that the currently annotated transcript catalogue consists of 17,525 (19,875-4,498 + 2,148) protein-coding genes if paralogs are counted only once. Of those, 19.1% (2,148 + 1,249) showed evidences of multiplication, whereas 90% (4,134 + 2,274) of the 7,117 (4,498 + 2,619) paralogs occurred as pairs. The remaining 709 (364 + 345) paralogs were grouped in 193 paralog families consisting of 3 and more paralogs (average: 4 paralogs per family, maximum: 23).

### *N. furzeri* transcriptome browser - NFINtb

A public web site interface named the “*Nothobranchius furzeri* Information Network transcriptome browser” (NFINtb, https://gen100.imb-jena.de/EST2UNI/nfintb/, Additional file 5: Figure S3) was set up. It provides the annotated transcript catalogue, including detailed BLAST results, predicted protein domains, associated gene ontology terms, microsatellites and gene expression data. Further information on sequenced libraries, distribution of contig lengths and annotation numbers, respective GO terms and corresponding transcript contigs is given. A query mask allows keyword (e.g. annotation, gene symbol, GO category) and BLAST searches by sequence similarity. *N. furzeri* sequences can be retrieved by batch download including annotation details.

### Differentially expressed genes in old *vs.* young *N. furzeri*

Subsequently to the completion of the first version of an *N. furzeri* transcript catalogue, selected RNA-seq datasets (libraries 6–13, Table 1) were used to provide a first transcriptome-wide insight into ageing of *N. furzeri*, i.e. transcript levels in skin and brain of young and old GRZ (5 and 14 weeks, respectively) and MZM-0403 (5 and 31 weeks, respectively) were analysed. For mapping of the reads, the longest transcript contig per annotated *N. furzeri* gene was used (19,875 contigs, 53 Mb). More than 17,000 of those genes were found to be expressed in the samples (Table 5).

**Table 5:** Summary of RNA-seq analysis

	**GRZ**				**MZM-0403**			
**Library**	**6**	**7**	**8**	**9**	**10**	**11**	**12**	**13**
Age (weeks)	5		14		5		31	
Organ	Skin	Brain	Skin	Brain	Skin	Brain	Skin	Brain
Reads	33,947,044	36,117,265	35,963,921	27,369,310	31,727,935	34,720,289	30,268,649	31,423,327
Mapped / Unique (%)	58 / 46	42 / 37	53 / 43	41 / 36	65 / 52	54 / 48	64 / 53	52 / 46
Transcribed genes	17,350	17,638	17,456	17,535	17,170	17,590	17,375	17,644
Median transcript level (rpkm ^a^)	2.3	4.7	2.6	5.7	2.5	5.4	3.5	5.8
Range of transcript levels (rpkm)	0.005 – 13,043	0.006 – 40,801	0.005 – 39,533	0.01 – 5,587	0.006 – 10,409	0.003 – 5,288	0.005 – 3,878	0.009 – 3,946

^a^ Reads per Kilobase per Million mapped reads.

**Figure 3:**
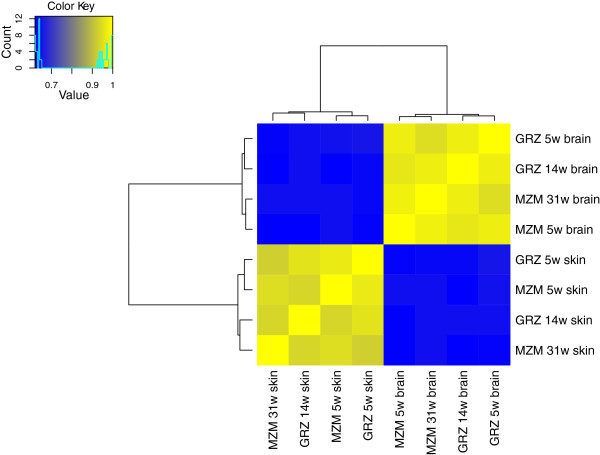
**Cluster heat map of *N. furzeri *transcript-level data.** Correlation analysis of eight *N. furzeri* RNA-seq datasets, which were derived from skin and brain of young and old specimens of the short-lived strain GRZ and the longer-lived strain MZM-0403. Young age represents 5 weeks in both strains, whereas old age is reached at 14 weeks in GRZ and 31 weeks in MZM-0403. Analysis is based on Spearman correlation coefficients of log-transformed RPKM transcript levels. Tissues show the highest correlation, irrespective of age and strain.

**Figure 4:**
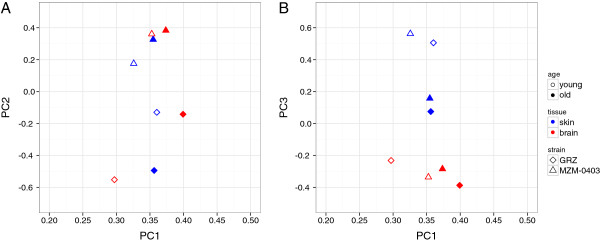
**Principal Component Analysis of transcript levels.** PCA results for all *N. furzeri* RNA-seq datasets are shown as biplots of the first *vs.* the second (**A**) and first *vs.* third component (**B**). The first component preferentially reflected the variation introduced by age whereas second and third components represented differences in strain and tissue, respectively.

Correlation of all data showed that samples from the same tissue were best correlated (0.93 to 0.96, Spearman correlation) (Figure 3). In skin, samples of the same age clustered better than those of the same strain, whereas in brain, samples of the same strain seemed to cluster better than age. Further, a principal component analysis (PCA) was performed (Figure 4). Eight principal components were identified, of which the top three explained 96% of the total variation in all samples (PC1: 56%; PC2: 22%; PC3: 18%). In the two-dimensional spaces spanned by the components, the first component preferentially reflected the variation introduced by age whereas second and third components represented differences in strain and tissue, respectively.

To identify genes statistically significant differentially expressed with age, the available RNA-seq datasets were lacking biological replicates as the primary goal of these sequencing efforts was the completion of the transcript catalogue (3–5 specimens were pooled before sequencing, Table 1). To overcome this limitation, we decided to abstract from strain-specific differences and used the respective datasets of the two *N. furzeri* strains as replicates. In total, 43 differentially expressed genes (DEGs) were found in each of the two tissues, respectively (p ≤ 0.01, DESeq [26]; Additional file 6). In skin, 11 genes were up- and 32 genes downregulated with age. In brain, there were 24 up- and 19 downregulated genes. The intersection of the DEG sets contained only *VDRB* (*vitamin D receptor b*) which was upregulated in both tissues.

To identify biological functions potentially affected by the changes in transcript levels, DEGs were submitted to the Database for Annotation, Visualization and Integrated Discovery (DAVID, [27,28]). DEGs were analysed for shared gene ontology terms, pathways and protein domains, and resulting enriched gene groups (minimum enrichment score ≥ 1) were considered. The 34 upregulated genes grouped into the following three annotation clusters: “activation of immune response”, “cell adhesion at the plasma membrane” and “positive regulation of apoptosis”. The first cluster was identified by genes upregulated in brain whereas the others contained genes from both tissues. The 51 downregulated genes seemed to act mostly as “collagens/proteoglycans” and were located in the “extracellular region”; these functional clusters were identified by skin DEGs only. Additional annotation clusters of downregulated genes from both tissues described processes like “cell cycle”, “cell division” and “cell proliferation” as well as “DNA replication” (Additional file 7).

**Figure 5:**
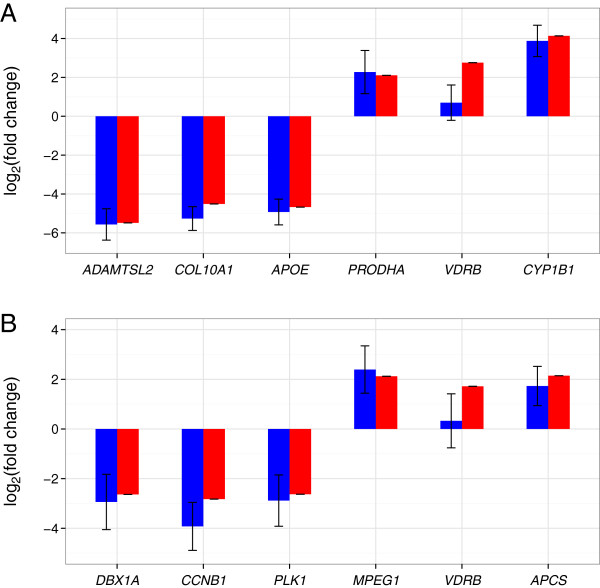
**Validation of selected DEGs by qRT-PCR.** Three up- and downregulated genes detected by RNA-seq (blue) were selected from skin (**A**) and brain (**B**), respectively, and qRT-PCR (red) was performed on the same RNA-samples. Except for *VDRB*, fold changes (log-transformed) did show good agreement between RNA-seq and qRT-PCR.

To verify RNA-seq data, three up- and downregulated genes were selected based on their annotated function from each tissue and analysed by quantitative reverse transcription PCR (qRT-PCR, Figure 5, Additional file 8: Table S2). Except for *VDRB*, qualitative changes in transcript levels were confirmed, with quantitative values being in good agreement.

To evaluate whether the observed *N. furzeri* ageing signature is specific to this species or more general for fish, we performed a similar analysis in zebrafish which serves a model organism for studying many aspects of biology, e.g. ageing. To consider in this case biological replicates from the beginning, we generated RNA-seq data from skin of 5 and 42 month old individual zebrafish (10 and 5 specimens, respectively). In total, 1,191 zebrafish DEGs were found (p ≤ 0.01). This number is much higher than in *N. furzeri*, which is likely due to the presence of a more appropriate selection of biological replicates. Of the 43 DEGs identified in *N. furzeri* skin, 19 (43%) were also present in zebrafish DEGs and showed the same direction in fold change (Additional file 9). This was significantly different from chance (p = 2.4x10^-14^, binomial test). Of the 24 remaining genes, additional 16 (33%) showed the same direction, but did not reach significance in zebrafish. One of the remaining eight genes (*cytokine-like 1*, *CYTL1*) was significant in both species with a different direction (*N. furzeri*: downregulated, zebrafish: upregulated). To summarise, we found that a significant number of DEGs was similarly regulated in ageing of *N. furzeri* and zebrafish.

### Strain-specific signatures of ageing in *N. furzeri*

**Figure 6:**
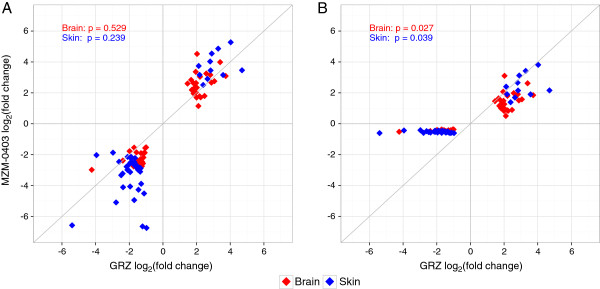
**Fold change differences between DEGs of the short- and the longer-lived *N. furzeri *strains.** A scatterplot of fold changes (log-transformed) in the short-lived strain GRZ and the longer-lived strain MZM403 is shown. The 43 DEGs in skin are given in blue, and the 43 DEGs in brain in red. In (**A**), fold changes are shown as determined over the time span of 9 and 26 weeks for GRZ and MZM-0403, respectively. In (**B**), MZM-0403 fold changes were normalised to the GRZ time span of 9 weeks. P-values are calculated to test for differences in fold changes between GRZ and MZM-0403 using two-sample, paired Wilcoxon test. In contrast to standard fold changes (**A**), time-normalised fold changes (**B**) were significantly different between GRZ and MZM-0403 in skin and brain.

To detect ageing-related differences between the short- and the longer-lived *N. furzeri* strains, we considered strain-specific differences in fold changes of the 43 DEGs identified in skin and brain, respectively. There was a trend towards larger differences in the longer-lived strain MZM-0403 (Figure 6A), although not statistically significant (brain: p = 0.529, skin = 0.239; two-sample, paired Wilcoxon test). However, these fold changes were calculated using the transcript levels at 5 weeks, i.e. when both strains reach sexual maturity, as baseline, whereas chronological age at the second time point differed between the strains, i.e. old age is reached at 14 weeks in GRZ but at 31 weeks in MZM-0403. To account for this difference and simplistically assuming linear changes in transcript levels, fold changes of the longer-lived MZM-0403 at 31 weeks were scaled down to an age of 14 weeks (Figure 6B). Accordingly, normalised fold changes were significantly different between GRZ and MZM-0403 in skin (p = 0.038) and brain (p = 0.026). When up- and downregulated genes were analysed separately, this was also significant for brain (p = 9.1x10^-5^ and 3.8x10^-6^, respectively) and partly significant for skin (p = 0.067 and 2.3x10^-10^). In conclusion, fold changes over chronological time were more pronounced for GRZ than MZM-0403, which suggested that ageing is accelerated in GRZ.

## Discussion

We built a comprehensive, annotated transcript catalogue for *N. furzeri*, the vertebrate species with the shortest lifespan recorded for vertebrates in captivity, and identified 85 genes showing significantly changed transcript levels over the *N. furzeri* lifetime.

### Developing an assembly strategy

Transcriptome assembly is still a challenging task, mainly because most available tools are designed for assembling genomes and therefore assume an even coverage distribution and error rate [29]. Transcript levels, however, are highly variable, which results in an uneven read coverage distribution and a variable error rate. Also, genome assembly usually is aimed at generating one consensus sequence, whereas in transcriptome assembly, alternative splicing can lead to multiple consensus sequences for one gene. Further challenges are introduced when both classical and next-generation sequencing technologies are employed, because these differ in sequencing coverage, read length and accuracy.

We addressed these problems by applying a two-step strategy. First, Sanger and 454/Roche reads were assembled using PAVE, a classical overlap-layout-consensus assembly tool. PAVE compensates well for sequencing errors and less perfect overlaps and yields longer contigs within a reasonable runtime. Second, Solexa/Illumina reads were iteratively assembled onto a backbone of PAVE contigs using a de Brujin graph assembly tool, which is better suited for large amounts of short reads and much faster than PAVE. Two additional steps were of importance for the iterative assembly procedure. One, input complexity of each next-generation library can be largely reduced by mapping reads onto existing contigs and discarding those reads that fully align to a contig. Two, since de Brujin graph assembly tools are more susceptible by sequencing errors and thus tend to assemble shorter contigs, a classical overlap-layout-consensus assembly tool was used to merge these contigs into larger ones.

### Quality assessment of the transcriptome assembly

Quality control of assemblies is essential because the quality of the assembled contigs has a direct impact on subsequent analyses. However, while a number of established metrics and protocols exist for genome assembly, only a few have been recently proposed for transcriptome assembly [29,30]. Moreover, they were originally defined for reference-based transcriptome assemblies and require a genome sequence to compare against; although, technically, a set of already assembled transcript sequences might also serve as reference. This is often not the case for *de novo* transcriptome assemblies and makes quality assessment much more difficult. In case of *N. furzeri*, only very few sequences were available, and their use as reference was prevented by their small number. Instead, we used a set of protein sequences with experimental support from medaka, the closest relative of *N. furzeri* with a genome available.

The majority of these medaka proteins (74%) were hit by at least one *N. furzeri* transcript contig, which suggested a good representation of these proteins in the transcript catalogue. Moreover, most proteins were completely covered by *N. furzeri* transcript contigs and, notably, a large number of proteins were completely represented by a single contig. Note that these results were obtained from a cross-species comparison, and the sequence divergence between the two species might interfere with the results, thus representing a conservative estimate.

The remaining proteins without hits might be missed for several reasons. First of all, the corresponding genes might not be expressed in the sequenced transcriptomes. Alternatively, the sequence divergence between the *N. furzeri* and medaka proteins might be too high so that BLASTx failed to identify these proteins. Finally, their genes might not be present in *N. furzeri* or were turned into pseudogenes without expression.

We also assessed the fragmentation and redundancy of the transcript catalogue. The fragmentation was relatively low, and only a few transcript contigs were regarded as fragments (non-overlapping). In contrast, the vast majority of the medaka protein sequences were covered by multiple contigs, indicative for alternative splicing and/or gene duplication events.

Chimeric transcript contigs may originate from gene or transcript fusions and assembly errors. The observed low putative-chimerism rate of 0.3% was regarded as an indication for a negligible misassembly rate not requiring further computational and / or experimental efforts. More insights into the falsity or validity of these putative chimeric transcript contigs will provide an *N. furzeri* genome sequence in the future.

### Completeness of the transcript catalogue

The presented *N. furzeri* transcript catalogue represents almost 20,000 protein-coding genes, the number typically expected for a vertebrate genome [31]. However, without a high-quality reference genome sequence, it is not possible to determine the true number of *N. furzeri* genes. Therefore, we first compared the gene number to respective numbers in fish species with a sequenced genome, i.e. medaka, stickleback, tetraodon and zebrafish. Hits to annotated genes indicated that between 71% and 87% of *N. furzeri* protein-coding genes were identified by our approach. But comparing gene numbers between species heavily depends on the quality of annotations. Therefore, we determined gene families since their number should be more constant among fish. The results of this analysis led to the conclusion that the presented *N. furzeri* transcript catalogue is in terms of completeness comparable to gene catalogues of model fish for which genomes have been sequenced.

The genes missing in our *N. furzeri* transcript catalogue are likely those expressed in early development of *N. furzeri*, because we used specimens of at least one week of age and were not able to include embryonic tissue. For example, in zebrafish, previous studies isolated several hundred genes involved in developmental control [32,33]. Additionally, gene annotation in zebrafish is currently far more comprehensive than in the other three fish species medaka, stickleback and tetraodon, and is also largely supported by expression data. Thus, it is most likely that a number of genes remain to be identified also in the other species. In addition, the number of gene copies retained after the fish-specific genome duplication varies considerably between the four species [34]. Most likely, this is also the case for *N. furzeri*. Thus, we cannot exclude that some of the missing genes are actually lost in *N. furzeri*.

In addition to overall gene number, the representation of CDS is important. For more than 70% of the *N. furzeri* protein-coding genes, a transcript contig with a complete (> 90%) CDS is present. When the sum of the non-redundant CDS identified in the transcript catalogue was compared to the total CDS of all reference entries used for annotation, completeness was estimated to be 83%. Future analyses on alternative UTRs and splice isoforms will greatly benefit from the transcript catalogue once the *N. furzeri* genome sequence is available.

### Duplicated genes in *N. furzeri*

We found evidences for multiple copies of about 19% of the *N. furzeri* protein-coding genes, whereas the vast majority occurs in paralogous pairs reminiscent to the 3rd genome duplication in teleost fish [24,25]. Conversely, this indicates that >80% of gene copies originated by this genome duplication were lost from the *N. furzeri* genome which corresponds to previous estimates for teleost fishes of 76–85% [34]. Moreover, it has to be considered, that not all gene duplications may date back to the teleost-specific genome duplication and that by our transcriptome approach only paralogs could be detected whose expression was sufficiently high in the analysed samples. Once the *N. furzeri* genome sequence is available, a more comprehensive assessment of duplicated genes and their evolutionary fate will be possible.

### Detection of ageing-related, differentially expressed genes in *N. furzeri*

Selected RNA-seq experiments initially aimed at completing the transcript catalogue were subsequently further exploited to detect changes in *N. furzeri* gene expression over lifetime. For mapping and quantification of the respective datasets, only the longest transcript contig of each gene was used (19,875 contigs, 53 Mb). Respectively, only 53% of reads mapped uniquely to this reference. When using all transcript contigs with gene annotations as reference sequences (85,431 contigs, 144 Mb), mapping efficiency increased to 91%, thus indicating that initial mapping results are not due to incompleteness of the transcript catalogue. On the other hand, the latter results included a high fraction of multiple mappings (~80%). This redundancy of the transcript catalogue is caused both by the assembly of contigs representing alternative splicing events and gene paralogs as well by assembly problems due to sequencing errors and repetitive motifs. Solutions to deal with multiple mappings were described previously [35-38], but most are designed for genome reference sequences and presumably would introduce additional bias into our transcriptome data and thus were not applied. However, we did compare mapping efficiency using the zebrafish RNA-seq data. Interestingly, when we used transcript instead of genome sequences as a reference for mapping the zebrafish data, mapping percentages were between 40% and 49% vs. up to 80% for the genome (data not shown). This suggested that the number of mapped reads is largely determined by the respective reference, and using genome sequences as reference is superior to using all (known) transcripts. Again, it is highly desirable to generate an *N. furzeri* genome reference to shed additional light on this question.

Despite these limitations, we quantified transcript levels in skin and brain of young and old GRZ and MZM-0403. In all samples, over 17,000 genes (Table 5) could be profiled regardless of tissue. This was surprising, because transcriptome profiles are generally believed to be specific for the analysed tissue or organ [39-41]; however, many genes had very low transcript levels, suggesting that RNA-seq detected some kind of baseline transcription. When analysing the transcriptome profiles for general correlation, samples clearly clustered according to tissues. Interestingly, additional weaker but tissue-specific correlations were observed. For skin, samples of the same age clustered next to each other, indicating that age-specific transcript patterns are similar in both short- and longer-lived strains. In contrast, in brain samples of the same strain clustered next, suggesting that in this tissue transcriptome profiles differ more between strains than between ages. Finally, PCA conducted on all samples showed that most of the variation between the *N. furzeri* transcriptome profiles can be explained by age (reflected by PC1 which explains 56% of the variation), strain (PC2, 22%) and tissue (PC3, 18%). Notably, based on correlation, samples of the same tissue are most similar and, consequently, PCA identified tissue as the component that introduces the least amount of variation.

We identified 43 DEGs in *N. furzeri* skin and brain, respectively. Interestingly, these tissues differ in the major direction of transcript level changes with age. In skin, the majority of DEGs were down regulated whereas in brain the opposite was found. This observation is in good agreement with both correlation analysis and PCA and again points to tissue-specific signatures of ageing. Further, DEGs functions reflect expected signs of an ageing phenotype [42]. DEGs involved in apoptosis were upregulated whereas DEGs with a role in cell cycle control, cell division and proliferation were down regulated. This was, however, more prominent in brain than in skin, suggesting more dynamic/pronounced changes of transcript levels in brain over lifetime compared to skin. Transcript levels also showed an increase in inflammation in brain, which previously has been reported as another sign of age-related decline in brain [43,44]. In skin, a number of downregulated DEGs were involved in the maintenance of collagenous tissue and cartilage.

Unfortunately, the RNA-seq datasets for *N. furzeri* did lack replicates, as they primarily were designed to complete the transcript catalogue, i.e. represent pooled samples of 3–5 specimens. On the other hand, those pooled datasets are less prone to fluctuations. The validation of selected DEGs by qRT-PCR showed that the rate of putative false positives was low (2 of 12). As in both cases the same qRT-PCR assay failed to reach significance (although the direction of expression change was confirmed), a technical PCR problem cannot be ruled out. However, the present approach using only two datasets derived from different strains as biological replicates most likely missed a considerable number of DEGs that show higher variation within or between strains and therefore failed to reach significance.

Using RNA-seq data from skin in young and old zebrafish we were able to confirm that transcript patterns of ageing are similar in both species. Thus, *N. furzeri*, in spite of its exceptionally short lifespan of only few months, exhibits general characteristics of vertebrate ageing which is in line with previously observed typical cytological and physiological ageing-related phenotypes [10].

### Accelerated ageing in *N. furzeri* strain GRZ

Previous work based on the analysis of age-related biomarkers suggested that ageing is accelerated in the short-lived strain GRZ compared to the longer-lived strain MZM-0403 [7,45]. Our analysis of whole transcriptomes of GRZ and MZM-0403 also supported the difference in ageing pace between these strains. When comparing time-normalised fold changes of identified DEGs, we found that age-related transcript level changes are stronger in GRZ than in MZM-0403. As the assumption of linear changes in transcript levels with age is most likely in many cases an oversimplification, the presented two-point analysis is a first approximation. Further studies should include additional time points to better dissect chronological and biological ageing in the *N. furzeri* strains.

### Outlook

Future work will be directed towards completing the transcript catalogue, e.g. including genes expressed in early development. In parallel, we are working on the assembly and annotation of a first working draft of the *N. furzeri* genome sequence which will be available in the near future. Transcriptome and genome sequences will be used to determine gene structures, detect splice isoforms and genes encoding non-protein-coding RNA species. Second, further RNA-seq experiments will be performed utilising more and strain-specific biological replicates as well as additional time points to better understand ageing-related mechanisms in *N. furzeri* and vertebrates in general.

## Conclusions

We established a high-quality, comprehensive and annotated transcript catalogue of the short-lived fish species *N. furzeri*, a novel model organism for age research. We employed it as a mapping reference for RNA-seq experiments and identified genes which transcript levels were significantly changed upon ageing. Regulation was tissue-specific with the majority of genes upregulated in brain but downregulated in skin. A comparison to zebrafish suggested that transcript patterns of ageing are similar in both species. Further, ageing-related changes were accelerated in the short-lived *N. furzeri* strain with respect to the longer-lived.

## Methods

### Fish strains and maintenance

*N. furzeri* strains GRZ and MZM-0403 were described previously [7,9]. Up to 12 fish per strain were kept in 40 litre tanks at 26°C under a light regime of 12:12 h light:dark and fed on red mosquito larvae (*Chironomidae*) ad libitum once a day. Water was filtered using air-driven foam filters; the water was changed once a week. Wild-type zebrafish of the TüAB strain were kept in groups in an open circulating standard zebrafish system. All experiments were performed according to the “Principles of laboratory animal care” as well as to the current version of the German Law on the Protection of Animals.

### RNA isolation, library construction and sequencing

For *N. furzeri*, total RNA was isolated from skin and brain using the RNeasy Mini kit (Qiagen) and from whole body using the RNeasy Midi kit (Qiagen). Males and females were used as given in Table 1. For zebrafish, total RNA was isolated from skin of male specimens aged 5 and 42 months, respectively, using Trizol (Invitrogen).

For Sanger sequencing (library 1, Table 1), cDNA was normalised by Evrogen (Moscow, Russia) and cloned into pAL17.3 via SfiI. Recombinant plasmids were amplified in *E. coli*, purified, sequenced from both ends (BigDye Terminator v.2.1 Cycle Sequencing Kit, ABI, Weiterstadt, Germany) and separated on ABI 3730xl capillary sequencers.

For 454/Roche sequencing, normalised and non-normalised cDNA was prepared by Evrogen, Moscow, Russia (library 2 and 3, Table 1) and Vertis, Freising, Germany (library 4, Table 1). The Roche adaptor containing cDNA libraries were diluted and subjected to emPCR followed by sequencing according to the manufacturer’s instructions (Roche Diagnostics). Sequencing was performed on 70 × 75 picotiter plates by two and a half runs using the GS LR70 (FLX) Sequencing kit and two runs with the XLR70t (Titanium) kit, respectively.

For Solexa/Illumina sequencing, *N. furzeri* libraries 5 to 13 and zebrafish libraries were prepared using the mRNA-Seq 8 sample prep kit (RS-100-0801) (Illumina, San Diego, CA, USA) according to the manufacturer’s instruction. Clusters were generated using Illumina’s Single Read Cluster Generation Kit v4 (GD-103-4001) or Paired End Cluster Generation Kit v4 (PE-203-4001), respectively. Each library was loaded onto one lane of the flow cell at 7 pM concentration. Sequencing of *N. furzeri* and zebrafish libraries was performed on a Genome Analyzer IIx (Illumina) for either 76, 101 or 150 cycles (Table 1), using Illumina’s 36 Cycle Sequencing Kit v4 (FC-104-4002) following the manufacturer’s protocol.

Sanger reads were deposited at the NCBI dbEST database. 454/Roche and Solexa/Illumina reads were submitted to the NCBI sequence read archive (SRA) under submission accession SRA050046. A NCBI BioProject, accession PRJNA85613, provides links to all submitted sequence data. Zebrafish Solexa/Illumina reads were submitted to the NCBI SRA under submission accession SRA054207 (NCBI BioProject PRJNA169239).

### Read pre-processing

Sanger and 454/Roche reads with associated quality values were pre-processed as follows: Removal of known vector sequences and poly (A) tails as well as masking of low complexity regions was done with SeqClean (v. 1.0, [46]). Repetitive elements were masked with RepeatMasker (v. 3.2.9, [47]) and a library of *N. furzeri*-specific repeats [9]. For, Solexa/Illumina libraries, the String Graph Assembler (v. 0.9.12, [48]) was used to correct sequencing errors and, for assembly, to remove low-quality reads.

### Assembly

TGICL (v. 2.0), Newbler (v. 2.3) and PAVE (v. 2.0) were run with default parameters. Only contigs longer than or equal to 300 bp were further analysed. Solexa/Illumina libraries were assembled using the following iterative assembly procedure on each library: In each iteration, the Burrows-Wheeler Alignment tool (BWA v. 0.5.9, [16]) was used to map Solexa/Illumina reads of the respective library onto the previous assembly. Subsequently, remaining unmapped reads and contigs of the previous assembly were joined with the CLC Assembly Cell (v. 3.2.2, [49]), a de Bruijn graph assembly tool. To improve length and quality, the resulting contigs were further assembled with TGICL. To use large contigs (≥ 2,000 bp) as input for TGICL, they were split into 2,000 bp-fragments which overlap by 300 bp. After assembly, CD-HIT-EST (v. 4.5.5, [50]) was used to remove highly redundant contigs (99% identity and 90% coverage of the smaller sequence). Further, all contigs smaller than 300 bp were excluded. Each round resulted in a “new” assembly consisting of the previous one plus new sequence information introduced by the respective assembled Solexa/Illumina dataset and served as a backbone for the next assembly utilising the reads of a further library. Once this iterative assembly process was finished, *N. furzeri* GRZ Solexa/Illumina libraries were mapped with BWA onto previously generated contigs. The final GRZ-specific consensus sequence was called with SAMtools and BCFtools (v. 0.1.16, [51]) thereby improving the overall contig quality and correcting base calls introduced by MZM-0403 reads.

All transcript contigs were deposited at the NCBI transcriptome shotgun assembly (TSA) archive. The corresponding accessions are also available at the NCBI BioProject PRJNA85613 page.

### BLAST annotation

Annotation was based on BLAST searches against several protein and nucleotide databases (Table 3). BLAST parameters and e-value thresholds were defined for each database separately (Additional file 1: Table S1). Hits were filtered for maximum e-value, minimal overlap and minimum identity. Hits with non-informative descriptions like ‘bac’, ‘fosmid’ or ‘shotgun’ were excluded. Additional information like gene symbols or species names were parsed from definition lines of hits or, in case of Ensembl databases, fetched from the Ensembl MySQL server. Transcript contigs were annotated based on the best hits of each database and description, gene symbol and species name were copied as additional information.

### Gene ontology

Gene Ontology files (OBO v1.2) and database associations for UniProt were downloaded from the Gene Ontology web site [22] and stored in a MySQL database. GO terms were mapped to GO-Slim terms using the map2slim script (go-perl package, see Gene Ontology web site). For each transcript, UniProt identifier were parsed from its annotations and used to assign GO terms. For transcript contigs annotated with hits from Ensembl databases, additional GO terms were fetched from the Ensembl MySQL server.

### Identification of duplicated genes

To identify genes which are duplicated in teleost fish, the longest transcript contig per gene was compared using BLASTx (e-value ≤ 10^-07^) against Ensembl proteins of medaka, stickleback, tetraodon and zebrafish. Subsequently, information about paralogous genes in these fish species was obtained from the Ensembl Compara gene trees. In case, the two BLASTx hits point to Ensembl genes of two different species, the Ensembl gene of the first species is ‘replaced’ with the corresponding ortholog in the second species.

To identify genes exclusively duplicated in *N. furzeri*, all transcript contigs which belong to the same gene and share the same annotation were retrieved and translated into proteins. Only protein sequences with a minimum length of 100 aa were considered for further analysis. The protein sequence of the longest transcript contig was then aligned to each of the other (secondary) transcript contigs thereby generating pairwise protein alignments. Alignments were filtered for a minimum length of 100 aa and pairwise protein identities were calculated. The remaining (secondary) transcript contigs were clustered based on their protein divergence to the longest transcript contig with a minimum distance of 10% between each cluster. Two or more clusters indicate genes putatively duplicated exclusively in *N. furzeri*.

### Gene expression profiling and qRT-PCR validation

To quantify transcript levels in eight *N. furzeri* samples, 76 bp-reads from libraries 6–13 were mapped with BWA onto a nonredundant set of transcript contigs representing the longest transcript contig per gene. Zebrafish datasets were mapped with Bowtie [52] against the zebrafish genome (danRer7) and a precompiled exon junction database.

Only unique mappings were counted. For each gene, coverage was determined as number of reads mapping onto the respective transcript contig normalised to its length in kilobases and to the total number of mapped reads in millions (rpkm). Analyses of correlation (Spearman) and principal component analysis were done with R. Identification of DEGs was done with R and the DESeq package. Raw read counts were provided as input for DESeq.

qRT-PCR was carried out in MZM-0403. RNA of the same skin and brain samples analysed in the RNA-seq experiment was used. cDNA synthesis was performed in a 20 μl volume using 500 ng total RNA, 10 pmol Oligo-dT primer and 200 U SuperScript II reverse transcriptase (Invitrogen). Real-time PCR was performed with the SYBR GreenER qPCR SuperMix (Invitrogen) and using the iCycler iQ5 detection system (Bio-Rad). Primer sequences are given in Additional file 8: Table S2. Cycle threshold values were normalised to the gene *INSR* (insulin receptor) that already previously showed to be very stably expressed at different ages [53]. Fold changes were determined with the relative expression software tool [54].

## Competing interests

The authors declare that they have no competing interests.

## Authors’ contributions

AP performed transcriptome assembly and annotation, analysed RNA-seq data and set up the transcriptome browser. NH performed RNA isolation and qRT-PCR experiments. DS constructed normalised and non-normalised libraries. KR, MG and ST performed Sanger and next-generation sequencing. SP analysed the zebrafish data. MP, CE and KR conceived the study and participated in design and coordination. AP, KR and MP wrote the manuscript. All authors read and approved the manuscript.

## Supplementary Material

Additional file 1: Table S1 BLAST parameter adapted from [55]. Click here for file

Additional file 2: Figure S1 Functional annotation based on Gene Ontology. Second level GO-Slim terms for the longest transcripts per *N. furzeri* gene in the three domains: A) biological process, B) molecular function and C) cellular component. Click here for file

Additional file 3: Figure S2 BLAST analysis of *N. furzeri* transcript contigs *vs.* proteins of medaka, stickleback, tetraodon and zebrafish. Venn diagram showing 96,106 (46%) *N. furzeri* transcript contigs with BLASTx hits to Ensembl protein annotations of the four fish genomes. A set of 73,238 transcript contigs (35%) had hits in all four fish species, whereas 8,175 transcript contigs (4%) representing a total of 2,489 genes had hits exclusively in one species. Click here for file

Additional file 4 **Genes duplicated in *N. furzeri.*** List of duplicated genes identified in the *N. furzeri* transcript catalogue. Click here for file

Additional file 5: Figure S3 **NFINtb *N. furzeri *transcriptome browser.** To provide easy access, the *Nothobranchius furzeri* Information Network transcriptome browser was installed. The screenshot shows the query page, which is used to search transcript contigs by keywords (e.g. annotation, gene symbol, GO category). Click here for file

Additional file 6 ***N. furzeri *ageing-related genes in skin and brain.** Lists of differentially expressed genes in skin and brain of *N. furzeri* detected by RNA-seq. Click here for file

Additional file 7 **Enriched functional annotation terms obtained for *N. furzeri *ageing-related genes.** Enriched functional annotation terms obtained by DAVID for up- and downregulated genes as well as for skin- and brain-specific genes that were differentially expressed in *N. furzeri* with age. Click here for file

Additional file 8: Table S2 Primer sequences for qRT-PCR validation of RNA-seq results of selected *N. furzeri* ageing-related genes. Click here for file

Additional file 9 ***N. furzeri *ageing-related genes in skin and their confirmation in zebrafish.** Lists of *N. furzeri* differentially expressed genes in skin and their confirmation in skin of old vs. young zebrafish. Click here for file
