# Supplementary material for: The transcript catalogue of the short-lived fish Nothobranchius furzeri provides insights into age-dependent changes of mRNA levels
Source: BMC Genomics. 2013 Mar 16;14:185. doi: 10.1186/1471-2164-14-185 (PMC3605293; doi:10.1186/1471-2164-14-185)
Supplement: Additional file 1: Table S1 — BLAST parameter adapted from [55]. [file 1471-2164-14-185-S1.doc]

## Supplementary Table 1 – BLAST parameter adapted from [55]

| **Database** | **Program** | **Parameter** | **Max. e-value** |
| --- | --- | --- | --- |
| Ensembl fish proteins | wu-blastx | wordmask=seg lcmask W=4 T=20 | 10^-05^ |
| UniProt | wu-blastx | wordmask=seg lcmask W=4 T=20 | 10^-05^ |
| NCBI nr | wu-blastx | wordmask=seg lcmask W=4 T=20 | 10^-05^ |
| Refseq human proteins | wu-blastx | wordmask=seg lcmask W=4 T=20 | 10^-05^ |
|  |  |  |  |
| UniGene fish ESTs | wu-blastn | wordmask=seg lcmask M=1 N=-1 Q=3 R=2 W=9 | 10^-10^ |
| Ensembl fish transcripts | wu-tblastx | wordmask=seg W=4 T=999 hitdist=40 nogap | 10^-05^ |
