# Supplementary figures and images for: The transcript catalogue of the short-lived fish Nothobranchius furzeri provides insights into age-dependent changes of mRNA levels
Source: BMC Genomics. 2013 Mar 16;14:185. doi: 10.1186/1471-2164-14-185 (PMC3605293; doi:10.1186/1471-2164-14-185)

A

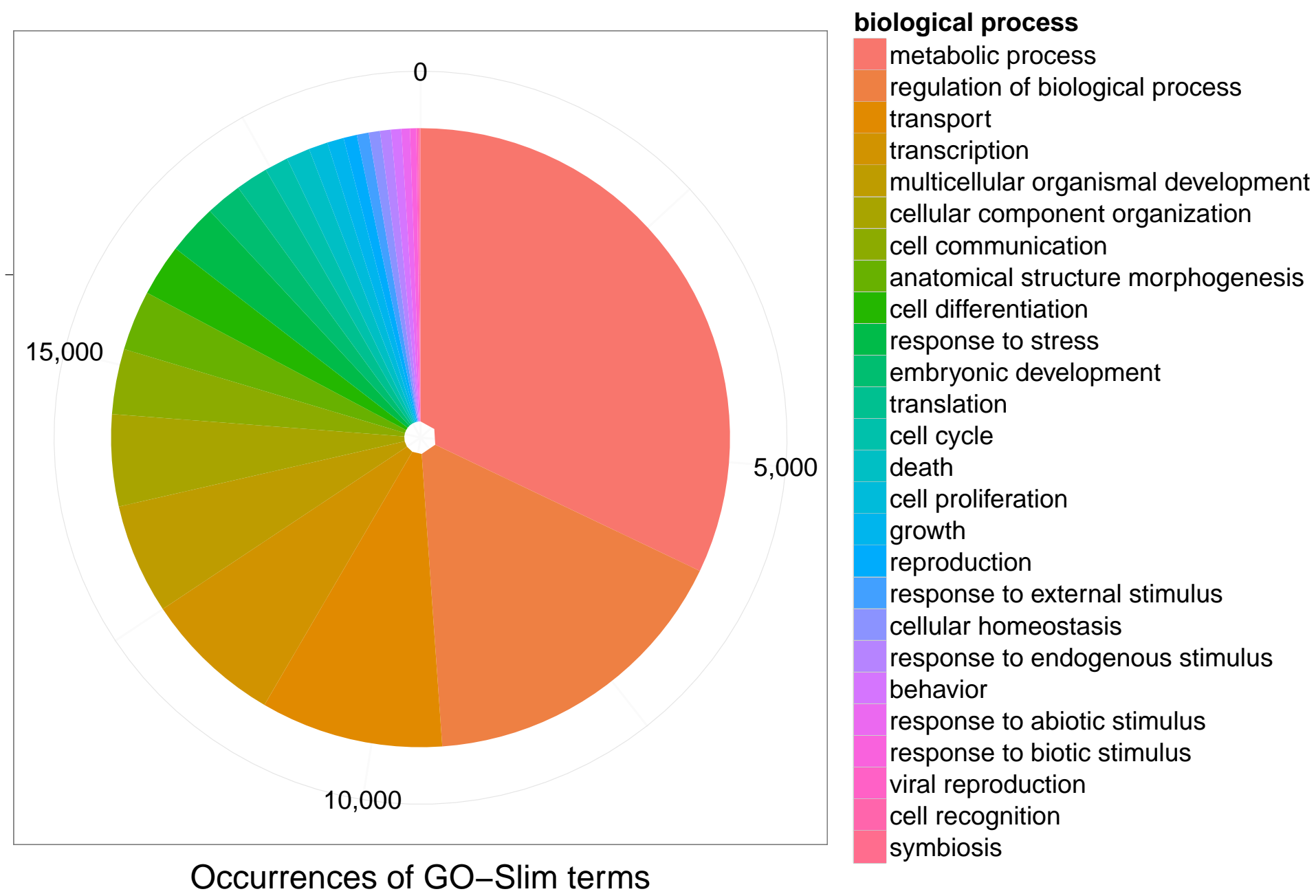

B

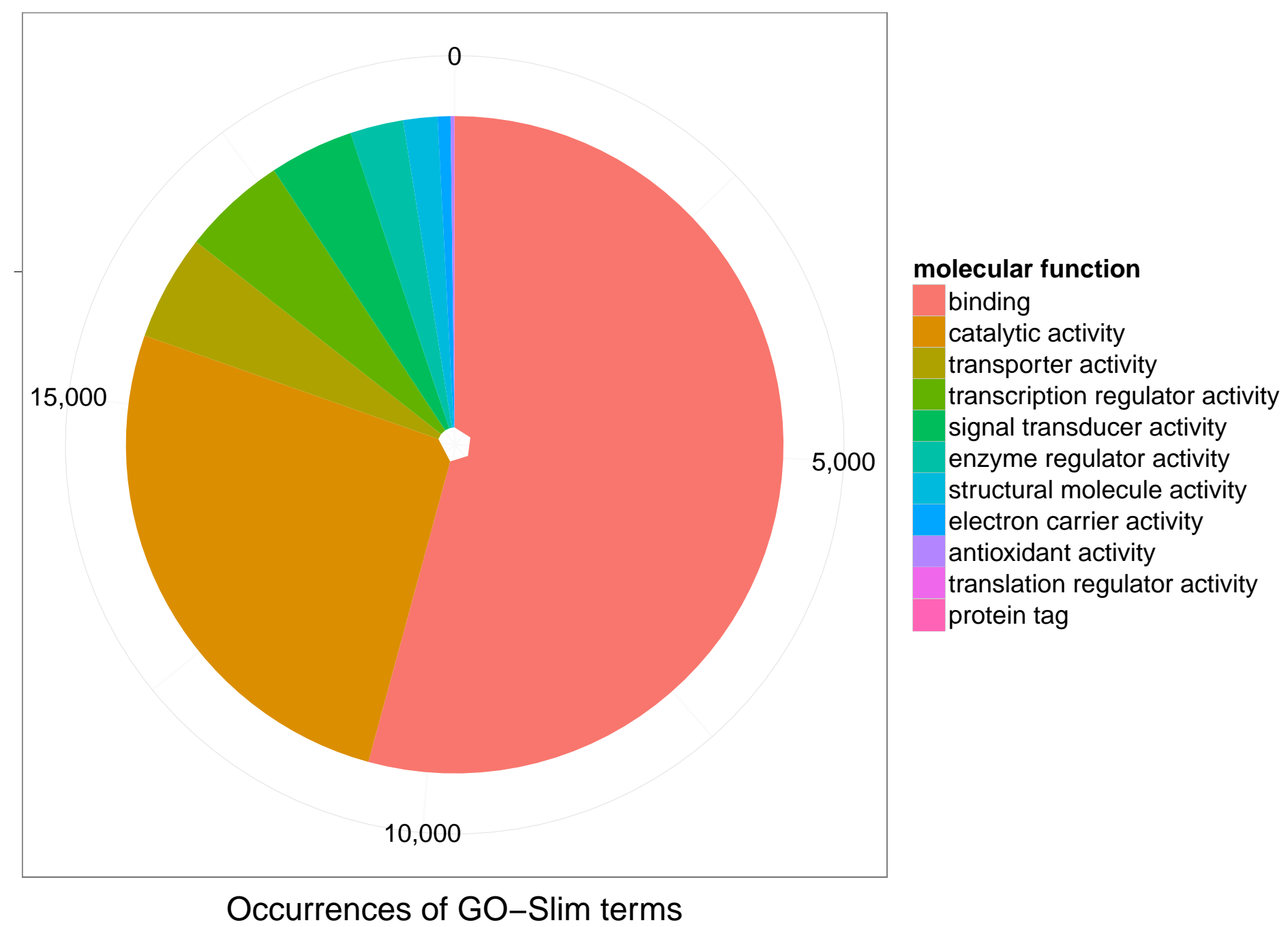

C

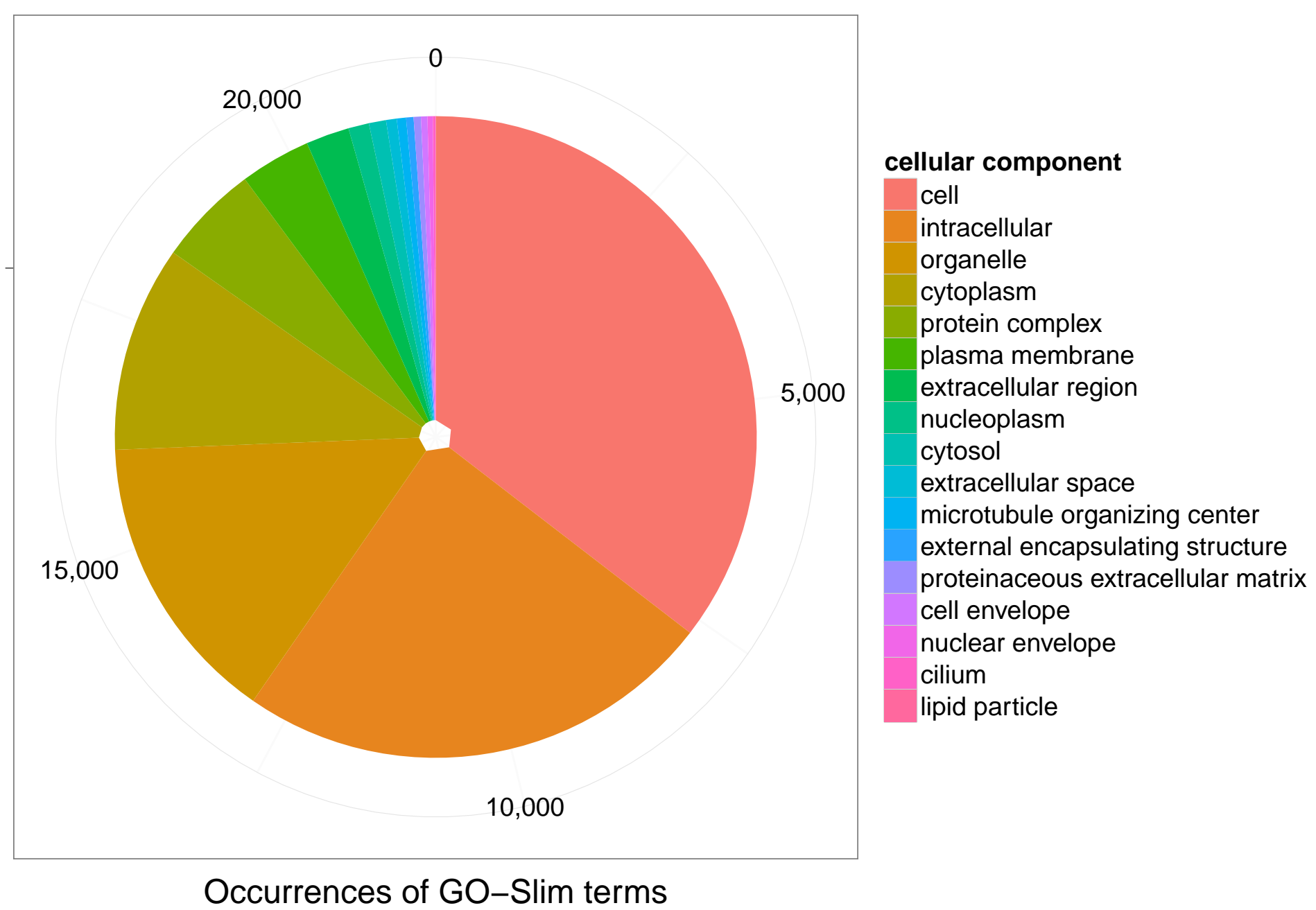

Supplement: Additional file 2: Figure S1 — Functional annotation based on Gene Ontology. Second level GO-Slim terms for the longest transcripts per N. furzeri gene in the three domains: A) biological process, B) molecular function and C) cellular component. [file 1471-2164-14-185-S2.pdf]

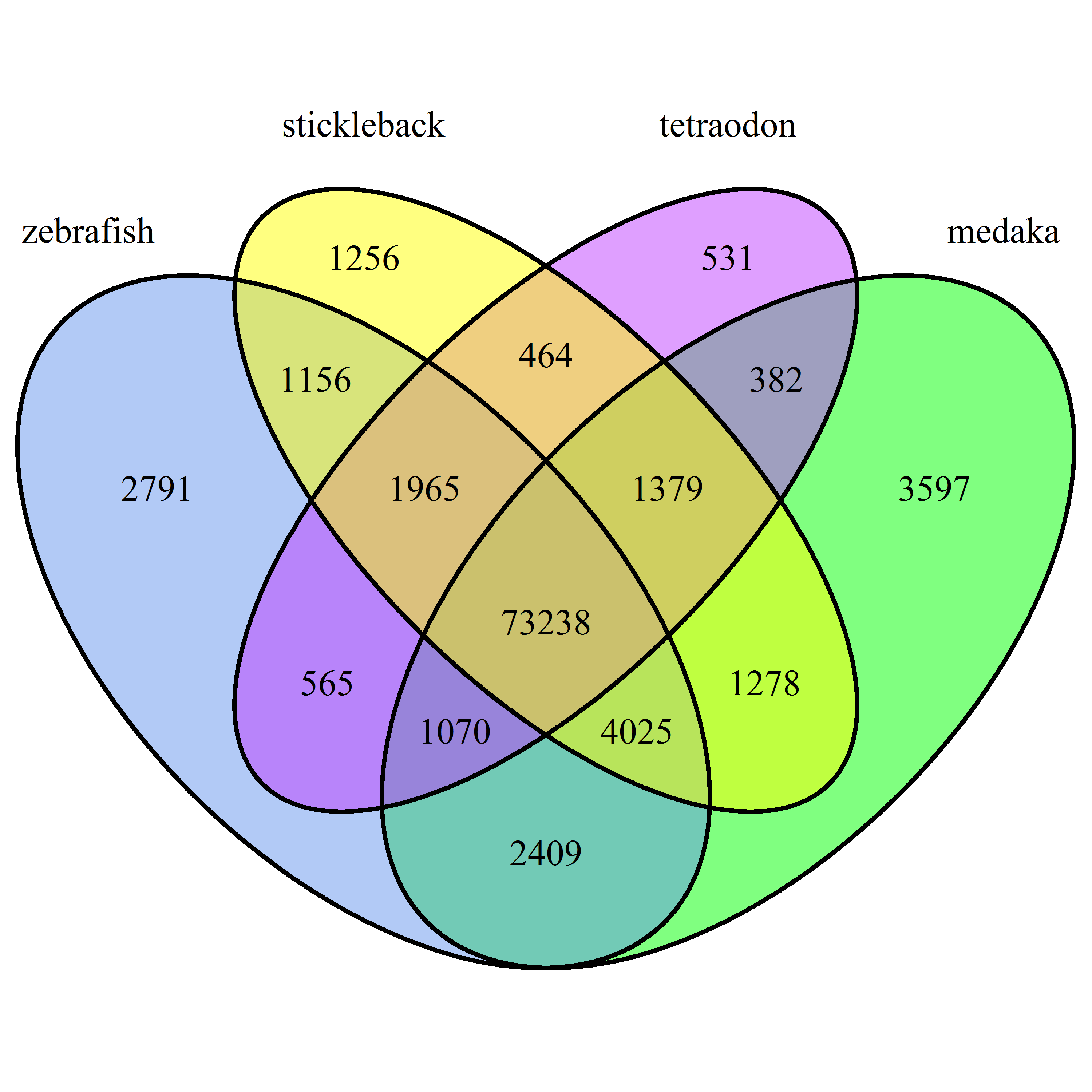

Supplement: Additional file 3: Figure S2 — BLAST analysis of N. furzeri transcript contigs vs. proteins of medaka, stickleback, tetraodon and zebrafish. Venn diagram showing 96,106 (46%) N. furzeri transcript contigs with BLASTx hits to Ensembl protein annotations of the four fish genomes. A set of 73,238 transcript contigs (35%) had hits in all four fish species, whereas 8,175 transcript contigs (4%) representing a total of 2,489 genes had hits exclusively in one species. [file 1471-2164-14-185-S3.tiff]

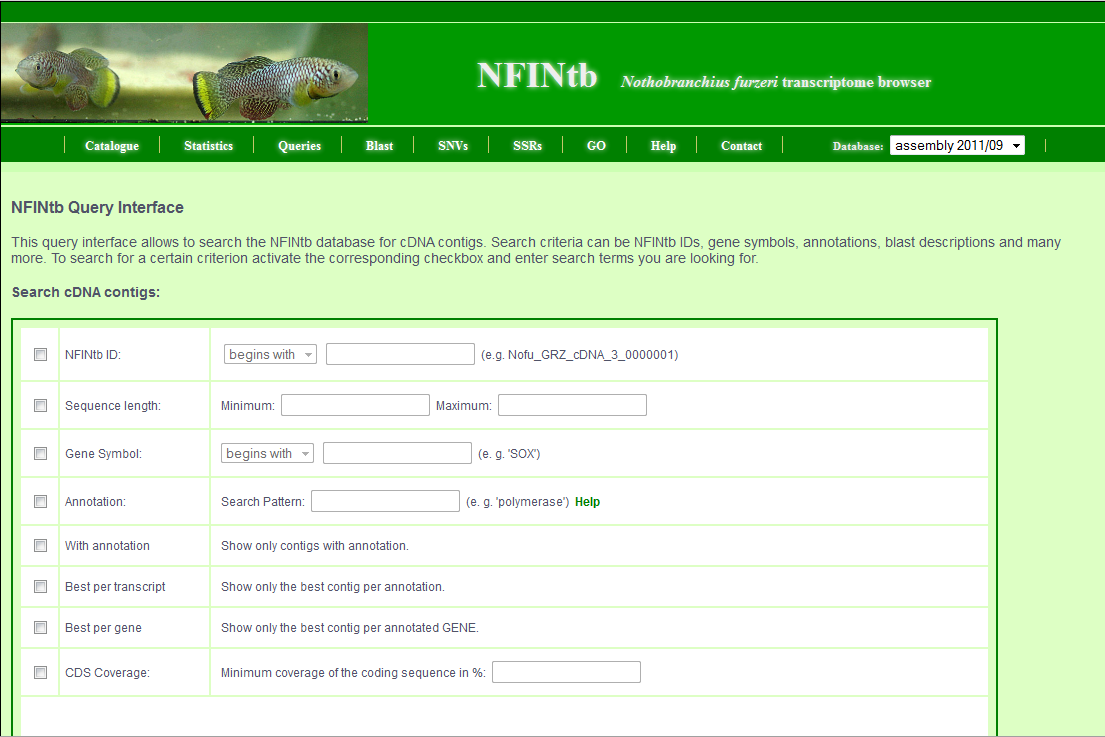

Supplement: Additional file 5: Figure S3 — NFINtb N. furzeri transcriptome browser. To provide easy access, the Nothobranchius furzeri Information Network transcriptome browser was installed. The screenshot shows the query page, which is used to search transcript contigs by keywords (e.g. annotation, gene symbol, GO category). [file 1471-2164-14-185-S5.tiff]
