# Supplementary material for: The transcript catalogue of the short-lived fish Nothobranchius furzeri provides insights into age-dependent changes of mRNA levels
Source: BMC Genomics. 2013 Mar 16;14:185. doi: 10.1186/1471-2164-14-185 (PMC3605293; doi:10.1186/1471-2164-14-185)
Supplement: Additional file 8: Table S2 — Primer sequences for qRT-PCR validation of RNA-seq results of selected N. furzeri ageing-related genes. [file 1471-2164-14-185-S8.doc]

## Supplementary Table 2 – Primer sequences for qRT-PCR validation of RNA-seq results for selected *N. furzeri* ageing-related genes

| **Gene** | **Annotation** | **Forward primer** | **Reverse primer** |
| --- | --- | --- | --- |
| ***INSR*** | insulin receptor | TGCCTCTTCAAACCCTGAGT | AGGATGGCGATCTTATCACG |
| ***ADAMTSL2*** | ADAMTS-like 2 | GCAGGCCTTGCTGTAGTACC | AAACCGGTGTCCAAACAGAC |
| ***APOE*** | apolipoprotein E | GCATAAGGACACCCAGGAGA | GGAGCAGGTCATTCAGGGTA |
| ***DBX1A*** | developing brain homeobox 1a | CATCAGCAAGCCAGACAGAA | GACATCCACCGGATGACAG |
| ***CCNB1*** | cyclin B1 | CCGTCACATAGGCAAAGTCC | CTGCTGCAGGAGACCATGTA |
| ***PLK1*** | polo-like kinase 1 | TGTGTTTGTGGTCCTGGAGA | TTGCCCAGTTTCAGGTCTCT |
| ***PRODHA*** | proline dehydrogenase (oxidase) 1a | GTGGATGCAGAGCAGACGTA | CCAAAATACCAGCCTTCTCG |
| ***VDRB*** | vitamin D receptor b | CATGCAGACTCAAACGCTGT | CGTGCTTCCTTTTCTGCTTC |
| ***CYP1B1*** | cytochrome P450, family 1, subfamily B, polypeptide 1 | CGGACATATTTGGAGCCAGT | AGCTGTTGCTGGTCTTCGAT |
| ***MPEG1*** | macrophage expressed gene 1 | CAGAAAAGCACCACAGCTCA | GGCCTTCGCTGTGTACATAAA |
| ***APCS*** | amyloid P component, serum | GAAGCTGGTCTGGTCCATGT | TCTGGAGCAAACATCACAGG |
| ***COL10A1*** | collagen, type X, alpha 1 | CCACTGGAAAGGGGTATGTG | GGCAGACCAATTCCATTCTC |
